# Supplementary material for: Implementation strategies and outcomes of intravenous iron use for treatment of anaemia during and after pregnancy in low- and middle-income countries: A scoping review
Source: PLOS Glob Public Health. 2026 Jan 9;6(1):e0004858. doi: 10.1371/journal.pgph.0004858 (PMC12788625; doi:10.1371/journal.pgph.0004858)
Supplement: Attachment — Submitted filename: Response to Reviewer Letter_PLOS GPH.docx [file pgph.0004858.s004.docx]

**Response to Reviewers: Manuscript ID: PGPH-D-25-01513**

**Implementation strategies and outcomes of intravenous iron use for treatment of anaemia during and after pregnancy in LMICs: a scoping review**

The Editor in-Chief

PLOS Global Public Health

Date: 10^th^ October, 2025

Dear Editor and Reviewers,

Thank you for the feedback on our manuscript. Please see below our point-by-point responses to the review comments.

Dr. Mobolanle Balogun, on behalf of the authors.

**REVIEWER 1**

1. The search strategy to begin with itself is very incomplete where the important terms to check implementation strategies or outcomes were not looked for. This has resulted in a very vague identification of studies which were designed to look for clinical outcomes than implementation outcomes. How can formative study describing barriers and facilitators be considered as implementation strategies...these would provide basis for developing implementation strategies which are completely not available nor described. The authors understanding on implementation research sounds quite good. However, they have missed the very essence in their search strategy.

**RESPONSE:** Thank you so much for these important points. Our considerations not to include implementation strategies of implementation outcomes in our search was not to narrow the search and miss out on studies, which for example set out to assess clinical outcomes and in the course of their study actually use implementation strategies or assess implementation outcomes such as acceptability or fidelity. As predicted, we found this to be the case during the review process. We had given our justification for this approach under the search strategy in the methodology.

2. There is no information about baseline situations in the studies in terms of availability of IV Iron type

**RESPONSE:** Thank you for your important comment. In the eligibility criteria stated in the methods we explained the concept of the scoping review; “This scoping review included studies that use IV iron of any brand, either alone or in comparison with other forms of treatment of anaemia in pregnancy and/or postpartum…” That implies that the studies concerned must have IV iron available to be able to use it. We also presented the types of IV iron in our results (Table 1).

3. The strategies described are inadequate to make any meaningful conclusions for eg. change in infrastructure - what change was done, at what level? whether this change was specific to providing IV Iron ?

**RESPONSE:** Thank you for your important comment. The concept of the study reads; “The studies proposed or described implementation strategies and/or implementation outcomes in the use of the intervention.” Thus, whatever strategy was charted was applicable to the use of IV iron. We used the Expert Recommendation for Implementing Change (ERIC) compilation for standardization and synthesis of the implementation strategies, which is important for uniform understanding by a global audience. This is critical as implementation science efforts have been complicated by the use of inconsistent language and inadequate descriptions of implementation strategies in the literature (please see citations below). However, the specifics of the strategies before classification using ERIC are included in the data extraction template (additional file 3).

a. Powell, B.J., Waltz, T.J., Chinman, M.J. et al. A refined compilation of implementation strategies: results from the Expert Recommendations for Implementing Change (ERIC) project. Implementation Sci 10, 21 (2015). <https://doi.org/10.1186/s13012-015-0209-1>
b. Waltz TJ, Powell BJ, Matthieu MM, Damschroder LJ, Chinman MJ, Smith JL, et al. Use of concept mapping to characterize relationships among implementation strategies and assess their feasibility and importance: results from the Expert Recommendations for Implementing Change (ERIC) study. Implementation Science. 2015 Dec 7;10(1):109.
4. No mention of the timelines needed for implementation

**RESPONSE:** Thank you for this important comment. We are uncertain if you are referring to the duration of use of IV iron within the studies. If that is the case, the duration of time for usage of IV iron was not considered relevant to our objectives but rather the assessment of implementation strategies and/or outcomes. Other specifics of implementation such as frequency and dose of IV iron are included in the data extraction template (additional file 3).

5. There is a mention of cost of IV iron as a major barrier...which is known. What implementation strategies were undertaken to address this?

**RESPONSE:** Thank you for this important comment. Since we charted data only from included studies, it is impossible for us to described implementation strategies to address cost where these are not stated in the studies. These studies assessed costs as an outcome and thus were relevant for inclusion. They did not describe strategies to reduce cost so this was not included in the scoping review. However, in our discussion, we noted that using financial strategies would be critical if IV iron is to be used sustainably in LMICs.

6. This entire exercise needs to be repeated to clearly **justify the review based on the objectives the authors set out to achieve**

**RESPONSE:** Thank you for this comment. Our scoping review was conducted in accordance with the Joanna Briggs Institute (JBI) methodology for conducting scoping reviews, which we followed thoroughly (please see citation below). We also addressed our set objectives and where we couldn’t (describing how implementation strategies and outcomes vary according to the study population (pregnant and postpartum women) and setting), this was described as a study limitation in the discussion.

a. Peters MDJ, Marnie C, Tricco AC, Pollock D, Munn Z, Alexander L, et al. Updated methodological guidance for the conduct of scoping reviews. JBI Evid Synth. 2020 Oct;18(10):2119–26.

Overall, we thank you for your review of our manuscript and trust that the explanations we have given, have provided further clarity to the paper.

**REVIEWER 2**

Thanks for the opportunity to review this narrative review of implementation strategies and outcomes of intravenous iron during and after pregnancy in LMICs. I believe that this review serves an important function by compiling results on this topic in one place, especially as multiple trials of FCM IV iron in pregnancy and postpartum along with accompanying implementation research are ongoing in LMICs. I would like to offer the following comments for the authors’ consideration.

1. Title – consider spelling out LMICs in the title

**RESPONSE:** Thank you for your suggestion. We have spelled out LMICs in the title.

2. Line 22 – Removed “the” before postpartum.

**RESPONSE:** Thank you for picking that. We have removed “the” before postpartum on that line.

3. Line 31 – If this is a narrative review, what was the role of descriptive statistics? Was this used for categorization or counting?

**RESPONSE:** Thank you for this important question. Our paper is a scoping review and not a narrative review. A narrative review is a summary of previously published articles on a topic, with the possibility of introducing modifications. In general, traditional narrative reviews are informal and do not follow a set structure. Unlike narrative reviews, a scoping review is structured, rigorous, transparent, and reproducible. In a scoping review, descriptive statistics play a vital role by summarizing and organizing the data from the included studies, providing an overview of the literature's characteristics and trends, and facilitating the identification of research gaps. I have included some citations to clarify the differences between narrative and scoping reviews and to support the use of descriptive statistics in scoping reviews.

a. Tulandi T, Suarthana E. Narrative Reviews, Systematic Reviews, and Scoping Reviews. J Obstet Gynaecol Can. 2021;43(12):1355-1356. doi:10.1016/j.jogc.2021.08.002
b. Pollock D, Evans C, Menghao Jia R, et al. "How-to": scoping review?. J Clin Epidemiol. 2024;176:111572. doi:10.1016/j.jclinepi.2024.111572
4. Line 55 and 59 – Fatigue is mentioned on both lines. Suggest combining or mentioning in only one place.

**RESPONSE:** Thank you for this. Fatigue is mentioned only in one place now.

5. Line 67 – Women also stop taking oral iron for other reasons, including misconceptions, forgetfulness, challenges with getting adequate supply, and socioeconomic barriers.

**RESPONSE:** Thank you for pointing this out. We have now included other barriers to oral iron supplementation with a citation.

6. Line 342 – 42.9% would not be considered most. The authors may want to consider rewording this.

**RESPONSE:** Thank you for pointing this out. We have now reworded this sentence, expanding on it to write out all of the results in prose. Therefore, the word “most” has been deleted.

7. Lines 360-361 – Please check if part of this sentence is missing.

**RESPONSE:** We have rephrased this sentence to make it clearer.

8. Table 3 – This is a useful table. I have some suggestions about making it easier to read. In the types of implementation strategies column, the authors could consider making a heading for each category, especially those that repeat (e.g., Assess for readiness and identify barriers and facilitators), instead listing it several times. In the key findings column, consider using bullet points within each row (e.g., separate bullets for barriers and facilitators).

**RESPONSE:** Thank you for the suggestions to make this table easier to read. We have now made a heading for each category, removing duplications on the list and used bullet points in the key findings column.

9. Table 5 – Similar to my comment on Table 3, in the types of implementation outcomes column, the authors could consider having heading for each outcome (e.g., acceptability, fidelity, feasibility, cost) instead of repeating in each row. The second to last row of the table has the name of an author in the types of implementation outcomes column. For the fidelity (46) row, it’s hard to understand the results in the key findings column because we don’t know what the expected duration of infusion or number of days between doses was. Cost (56) – IV iron sucrose as bolus compared to what (e.g., how many doses for the IV infusion)?

**RESPONSE:** Thank you for the suggestions. We have now adjusted the table to be similar to Table 3 and removed the name of the author which was included in error. Regarding the fidelity row, the authors of that particular study did not state a benchmark for complete compliance. However, we have now benchmarked the findings against standard treatment with iron sucrose for moderate anaemia in pregnancy (as relevant to the study population), which is included as a table footnote. We are careful to report only on findings charted from the studies in the table.

For the study reporting on cost of IV iron sucrose in bolus compared to IV iron sucrose infusions, the authors conducted an observational study giving iron sucrose bolus at the dosage of 200mg on alternate days to a maximum of 3 doses over a week. They did not give IV iron sucrose as infusion, which was the standard practice to give multiple infusions to make up for the total dose calculated by the standard formula [2.4 x weight in kg (target Hb–actual Hb)]. As we can only report on findings a reported in the study, we have simply indicated “multiple doses” for IV iron sucrose dosage in the table.

10. Line 428 – What does SHPs stand for?

**RESPONSE:** It stands for skilled health personnel. This has been written out in full now.

11. Discussion – Consider shortening the discussion. It is quite long and could be trimmed without losing meaning.

**RESPONSE:** Thank you for your suggestion. We have now trimmed the discussion without losing meaning.

12. Line 490 – Would the authors consider different countries as different settings? If so, then there is some variability in the setting.

**RESPONSE:** Thank you for this question. As noted in the methodology, the consideration for settings ab initio was health facilities, the community or other settings and not different countries.

13. Figures 2-4 – It’s nice to have some figures, but the information across these three figures could be shared more succinctly in a single table. Also, I noticed in Figure 2 that this refers to articles rather than studies. In some cases, I believe that some of the articles from a country are part of the same study. Would it make sense throughout the paper to refer to the number of studies rather than the number of articles?

**RESPONSE:** Thank you for these suggestions. We have merged the information in Figure 2 (countries) into Table 1. To avoid having another table and further consideration that the information on targets of implementation strategies and outcome was not a lot, we have written out the results in Figures 3 and 4 in prose form and taken out the figures.

Regarding your point about studies and articles, three of the studies included in the review assessed different outcomes within the same large project, while another two studies assessed different outcomes within another project. We have included this information in the results and chosen to refer to them as studies in the paper.

Overall, we thank you for your extensive review of our manuscript.
