# Supplementary material for: Implementation strategies and outcomes of intravenous iron use for treatment of anaemia during and after pregnancy in low- and middle-income countries: A scoping review
Source: PLOS Glob Public Health. 2026 Jan 9;6(1):e0004858. doi: 10.1371/journal.pgph.0004858 (PMC12788625; doi:10.1371/journal.pgph.0004858)
Supplement: Attachment — Submitted filename: Reviewer Comments_Final.docx [file pgph.0004858.s005.docx]

**Implementation strategies and outcomes of intravenous iron use for treatment of anaemia during and after pregnancy in LMICs: a scoping review**

**Reviewer Comments**

This manuscript presents a scoping review focusing on implementation strategies and outcomes of intravenous iron use in the treatment of anaemia during and after pregnancy in low- and middle-income countries (LMICs). The current burden of anaemia and the efficacy of IV iron use are evident in the treatment of anaemia. The authors followed JBI guidelines for this review and covered a broad range of databases, to provide a good overview of implementation strategy and outcomes.

1. The introduction is elaborate and descriptive, covering the well-known epidemiological facts. However, implementation gaps/data gaps need to be addressed. Authors can summarize the epidemiological background, emphasizing the inadequacy of IV iron use, research gaps in implementation, and the relevance of this scoping review to the issue.
2. Although the authors acknowledge the lack of implementation research, authors can compare the implementation differences between HICs and LMICs and the urgency of evidence from LMICs.
3. Methodology needs to be clear. Authors must clearly specify all search deviations, including changes in search terms, modifications to inclusion criteria, and any changes in analysis.
4. Include a paragraph stating the impact of differences in the type of studies, like RCTs and registry-based observational studies, on the interpretation of implementation outcomes.
5. The review reports the use of the implementation science frameworks and strategies. However, the discussion lacks adequate coverage of systemic causes that impact implementation, such as funding obstacles, training deficiencies, and policy limitations. The structural factors that limit the implementation of IV iron use in LMICs need to be elaborated upon or discussed.
6. The manuscript could be strengthened by a more profound thematic or conceptual synthesis, such as mapping barriers to specific categories, identifying regional patterns, or examining health system settings. Can add a graph/flow-chart/visual representation that summarizes barriers/facilitators/outcomes and gaps in research.
7. Additional discussion on the use of IV iron in the postpartum period would strengthen the manuscript, especially since it is highlighted in the title. The postpartum period appears to be under-represented in the included studies, likely due to the limited evidence available in the current literature. This gap should be explicitly acknowledged, along with a brief discussion of its implications for research and policy.”
8. Numbers and Tables should be made more readable. Please give clear captions to some figures. Table 3 is congested and difficult to read.
9. Regarding the PRISMA Flow Diagram, please elaborate further on the pre-2023 vs. updated 2025 search and ensure that all numbers are balanced in both the text and Figure 1.
10. Most studies are reported from India. Studies from other LMICs reflect minimal research. Please add relevant studies from other LMICs.
11. Some minor typesetting mistakes need to be corrected. Use uniform terms to refer to IV iron.
12. There are some inconsistencies in the use of abbreviations (e.g., SHP, FCM).

**Overall Recommendation:**

Moderate Revision

This paper covers a critical issue. The search is thorough, and the methods are of accepted standards. Nevertheless, significant changes are necessary to enhance clarity, methodological transparency, level of synthesis, and overall scientific rigor.
