# Supplementary material for: Implementation strategies and outcomes of intravenous iron use for treatment of anaemia during and after pregnancy in low- and middle-income countries: A scoping review
Source: PLOS Glob Public Health. 2026 Jan 9;6(1):e0004858. doi: 10.1371/journal.pgph.0004858 (PMC12788625; doi:10.1371/journal.pgph.0004858)
Supplement: Attachment — Submitted filename: Response to reviewer 3 comments.docx [file pgph.0004858.s006.docx]

**Response to Reviewers: Manuscript ID: PGPH-D-25-01513**

**Implementation strategies and outcomes of intravenous iron use for treatment of anaemia during and after pregnancy in low- and middle-income countries: a scoping review**

The Editor in-Chief

PLOS Global Public Health

Date: 2^nd^ December, 2025

Dear Editor and Reviewers,

Thank you for the feedback on our manuscript. Please see below our point-by-point responses to the reviewer 3’s comments.

Dr. Mobolanle Balogun, on behalf of the authors.

**Reviewer Comments**

This manuscript presents a scoping review focusing on implementation strategies and outcomes of intravenous iron use in the treatment of anaemia during and after pregnancy in low- and middle-income countries (LMICs). The current burden of anaemia and the efficacy of IV iron use are evident in the treatment of anaemia. The authors followed JBI guidelines for this review and covered a broad range of databases, to provide a good overview of implementation strategy and outcomes.

1. The introduction is elaborate and descriptive, covering the well-known epidemiological facts. However, implementation gaps/data gaps need to be addressed. Authors can summarize the epidemiological background, emphasizing the inadequacy of IV iron use, research gaps in implementation, and the relevance of this scoping review to the issue.

*Response: Thank you so much for your comments. We have now summarized the epidemiological background between lines 54 and 60. We have also provided further emphasis on the inadequacy of IV iron in LMICs between lines 83 and 88 where we write: “Yet, its implementation in LMICs lags significantly behind high-income countries, where IV iron is used widely due to its accessibility, preference and acceptability, and inclusion in treatment guidelines (20–22).*

*This implementation gap is striking despite strong evidence supporting IV iron's efficacy, health systems in LMICs struggle to integrate it into routine care amid concerns about cost, accessibility and poorly funded health-care infrastructure and systems (23)”.*

*The research gaps in implementation were extensively captured in the introduction from lines 90 to 101. We wrote: “While clinical trials have demonstrated IV iron's effectiveness (24,25), they tell us little about the practical challenges of implementing it in resource-constrained settings. Implementation science offers a lens to examine this challenge (26). By studying the strategies that work (or do not) in getting evidence into practice, we can identify practical solutions. A recent mixed-methods systematic review identified some barriers to and facilitators of IV iron implementation in pregnancy (27). However, no previous review has systematically mapped how LMICs are implementing IV iron for maternal anaemia. Which implementation strategies are being used? What outcomes are being measured? These questions remain unanswered.”*

*The relevance of our scoping review was captured in lines 115 to 120, where we wrote: “This scoping review represents the first comprehensive effort to map implementation strategies and outcomes for IV iron in maternal care across LMICs. This would be an important first step in informing the objectives of future systematic reviews on the topic (31). Understanding how to effectively implement IV iron therapy could transform the treatment of maternal anaemia in resource-limited settings, potentially saving thousands of lives and improving countless more.”*

2. Although the authors acknowledge the lack of implementation research, authors can compare the implementation differences between HICs and LMICs and the urgency of evidence from LMICs.

*Response: Thank you for your suggestions. We have expanded on the implementation differences between HICs and LMICs in lines 83 to 85, where we write: “Yet, its implementation in LMICs lags significantly behind high-income countries, where IV iron is used widely due to its accessibility, preference and acceptability, and inclusion in treatment guidelines.”*

*The urgency of evidence from LMICs was captured in lines 86-100 and highlighted in response to Point 1 above.*

3. Methodology needs to be clear. Authors must clearly specify all search deviations, including changes in search terms, modifications to inclusion criteria, and any changes in analysis.

*Response: Thank you for your comments. Our search strategy was explained in the methodology. We did not have and changes in search terms. In lines 180 – 188, we wrote: “The search strategy was developed in consultation with an experienced medical librarian, which aimed to locate both published and unpublished studies. A combination of keywords from the research questions was used to develop a full search strategy for electronic databases.*

*Considering that many studies may not use implementation science terminologies since they may not be implementation science studies, our search string was only a combination of intravenous iron, pregnant women, postpartum, anaemia, low-/middle-income countries and the synonyms of these terms identified from the initial search in MEDLINE (see Additional file 2).” Similarly, we did not have any modifications to our eligibility criteria, which is detailed in lines 140 to 155.*

*The limitation to our analysis, where we were unable to map how implementation strategies and outcomes vary according to study population and setting as stated in our protocol (as most of the studies were among pregnant women and in health facility settings), was mentioned as a limitation in lines 526 to 530. We wrote: “We set out to map how implementation strategies and outcomes vary according to study population, setting, and study design. However, little or no variation was found in the study population and settings, as most of the studies were among pregnant women as opposed to postpartum women and in health facility settings. Thus, that research question could not be fully explored.”*

4. Include a paragraph stating the impact of differences in the type of studies, like RCTs and registry-based observational studies, on the interpretation of implementation outcomes.

*Response: Thank you for your suggestion. From lines 252 to 256, we described the characteristics of the study designs in our scoping review. We wrote: “Study design: This was grouped into experimental studies (RCTs and quasi-experimental studies) and observational studies (comparative study, implementation study, quality improvement study, formative qualitative study, prospective study, cross-sectional study, retrospective analysis of routine data, cost-effective analysis, and registry).” Our reporting of results was also based on this grouping as we described implementation strategies and outcomes stratified by study design.*

*In the discussion, we have emphasized the impact of differences in the type of studies in lines 428 to 432. We write: “The general lack of implementation strategy or implementation outcomes assessment in experimental studies designs in this review could result in delays to bridging critical implementation gaps in LMICs, particularly as they incur heavier resource investments than observational studies”*

5. The review reports the use of the implementation science frameworks and strategies. However, the discussion lacks adequate coverage of systemic causes that impact implementation, such as funding obstacles, training deficiencies, and policy limitations. The structural factors that limit the implementation of IV iron use in LMICs need to be elaborated upon or discussed.

*Response: Thank you for your suggestions. We mentioned funding obstacle to IV iron implemention in our discussion in lines 454 to 456. We wrote: “Using financial strategies would be critical if IV iron is to be used sustainably in LMICs. Providers and patients in Nigeria have expressed concerns about out-of-pocket costs of IV iron particularly among poor women.”*

*We have expanded on the training deficiencies in lines 456 to 459. We write: “Only one observational study reported ongoing training of skilled health personnel as a strategy. There are training deficiencies in LMICs and educational strategies can address misconceptions and propagate the safety profile awareness of new IV iron formulations.”*

*We have put more emphasis on structural factors and policy limitations in lines 446 to 448. We write: “Interventions can be adapted throughout the implementation process due to diverse contexts and needs, particularly in LMICs with weak health-care systems and lack of sustained political will.”*

6. The manuscript could be strengthened by a more profound thematic or conceptual synthesis, such as mapping barriers to specific categories, identifying regional patterns, or examining health system settings. Can add a graph/flow-chart/visual representation that summarizes barriers/facilitators/outcomes and gaps in research.

*Response: Thank you for these suggestions to strengthen our manuscript. We would like to explain that our objectives were not to map out barriers and facilitators; we pointed out the systematic review that had done this in the introduction. The barriers and facilitators mentioned in our paper are key findings from an implementation strategy used. Doing any conceptual synthesis on barriers and facilitators would not align with our objectives. We have already done conceptual mapping for implementation strategies and outcomes using relevant implementation science frameworks, as aligned with our objectives.*

7. Additional discussion on the use of IV iron in the postpartum period would strengthen the manuscript, especially since it is highlighted in the title. The postpartum period appears to be under-represented in the included studies, likely due to the limited evidence available in the current literature. This gap should be explicitly acknowledged, along with a brief discussion of its implications for research and policy.”

*Response: Thank you for catching this. You have made a very valid point and we have now included further discussion about the gap in IV iron implementation in the postpartum period in lines 511 to 517. We write: “Another gap in studies on IV iron administration for obstetric anaemia in LMICs is the limited implementation assessment of IV iron in the postpartum period. This fragile period is characterised by high burden of anaemia, high attrition from postnatal care services, and inadequate follow-up care by limited numbers of providers in LMICs. The context of IV iron implementation likely differs in the antenatal and postnatal periods, thus research and policies that emphasise IV iron implementation in the postpartum women are critical.”*

8. Numbers and Tables should be made more readable. Please give clear captions to some figures. Table 3 is congested and difficult to read.

*Response: Thank you for your comments. Based on Reviewer 2’s earlier suggestions, we have removed the figures in the review and replaced them with tables. In Table 3, we have used bullet points for the report under the key findings section, thus making it easier to read.*

9. Regarding the PRISMA Flow Diagram, please elaborate further on the pre-2023 vs. updated 2025 search and ensure that all numbers are balanced in both the text and Figure 1.

Most studies are reported from India. Studies from other LMICs reflect minimal research. Please add relevant studies from other LMICs.

*Response: Thank you for your suggestions. The PRISMA flow diagram 2020 for updated systematic reviews is the flow chart that allows for updating of a previously completed review (Source: Page MJ, et al. BMJ 2021;372:n71. doi: 10.1136/bmj.n71.) A look at that flow chart shows there is not much information from the previous review with a lot more focus on the updated review. This does not apply for our study, which is a new review. What we did was to update our search before completing the review. We have made this clearer in lines 178 to 180. We write: “The search was initially conducted between 30th June and 30th July, 2023 and updated between 30th May and 4th June, 2025, before completion of the review.”*

*Regarding the findings on more studies from India, that was what was generated from searching literature extensively, using our well-defined eligibility criteria.*

10. Some minor typesetting mistakes need to be corrected. Use uniform terms to refer to IV iron.

*Response: We have used uniform terms to refer to IV iron.*

11. There are some inconsistencies in the use of abbreviations (e.g., SHP, FCM).

*Response: SHP has been written out in full following suggestions by reviewer 2.*

*FCM has now been written out in full in Table 3 and line 422. The abbreviation is only maintained in Table 5 key findings and spelt out in the foot to the table, to maintain brevity within the table.*

**Overall Recommendation:**

Moderate Revision

This paper covers a critical issue. The search is thorough, and the methods are of accepted standards. Nevertheless, significant changes are necessary to enhance clarity, methodological transparency, level of synthesis, and overall scientific rigor.

*Response: Overall, we thank you for your extensive review, which has helped to improve paper.*
